# *C20orf20* (MRG-binding protein) as a potential therapeutic target for colorectal cancer

**DOI:** 10.1038/sj.bjc.6605500

**Published:** 2010-01-05

**Authors:** K Yamaguchi, M Sakai, T Shimokawa, Y Yamada, Y Nakamura, Y Furukawa

**Affiliations:** 1 Division of Clinical Genome Research, Advanced Clinical Research Center, Institute of Medical Science, The University of Tokyo, 4-6-1 Shirokanedai, Minato-ku, Tokyo 108-8639, Japan; 2 Laboratory of Molecular Medicine, Human Genome Center, Institute of Medical Science, The University of Tokyo, 4-6-1 Shirokanedai, Minato-ku, Tokyo 108-8639, Japan; 3 Department of Anesthesiology, School of Medicine, The University of Tokyo, 7-3-1 Hongo, Bunkyo-ku, Tokyo 113-0033, Japan

**Keywords:** C20orf20 (MRGBP), BRD8, colorectal cancer, cell proliferation

## Abstract

**Background:**

Colorectal cancer is one of the most common causes of cancer death worldwide. Using cDNA microarray containing 23 040 genes, we earlier investigated gene-expression profiles in 11 colorectal cancers for the purpose of better understanding of colorectal carcinogenesis as well as development of novel diagnostic and therapeutic strategies. MRG-binding protein (*MRGBP*) or *C20orf20*, encoding a subunit of TRRAP/TIP60-containing histone acetyltransferase complex, was up-regulated in the majority of colorectal tumours.

**Methods and results:**

The elevated expression of MRGBP was observed in colorectal cancer tissues by quantitative PCR as well as immunohistochemical analyses. *MRGBP* marginally expressed in normal vital organs. Notably, suppressed MRGBP expression by *MRGBP* short hairpin RNA inhibited proliferation of colorectal cancer cells. Yeast two-hybrid screening and subsequent immunoprecipitation analysis identified bromodomain containing 8 (BRD8) as an MRGBP-interacting protein. As RNA interference against *BRD8* also suppressed proliferation of colorectal cancer cells, BRD8 may be an important down-stream target of MRGBP.

**Conclusion:**

These results suggest that MRGBP has an important function in proliferation of cancer cells through the regulation of BRD8 and that MRGBP should be a novel therapeutic target for colorectal cancer.

Colorectal cancer is the second leading cause of cancer death in the United States, and its incidence rates are increasing in Japan. It is estimated that there are almost 500 000 colorectal cancer-related deaths every year in the world (Parkin, 2001). Although recent medical advances have improved the prognosis of patients with the disease, complete cure of patients with advanced tumour is far from satisfactory. In chemotherapies for advanced colorectal cancer, oxaliplatin/fluorouracil/leucovorin (FOLFOX) is an effective and well-tolerated regimen. Combination of targeted biological agents such as anti-epidermal growth factor receptor (EGFR) with FOLFOX have been reported to enhance the efficacy against EGFR-expressing metastatic colorectal cancer (Giantonio, 2006; Tabernero *et al*, 2007). This indicates that the use of rationally selected therapeutic agents will improve the treatment for advanced diseases and results in increase of cure rate and/or prolonged survival. Regarding colorectal cancer, combination chemotherapy with Bevacizumab, an inhibitor of VEGF receptor, was approved in the United States and was shown to be effective for 45% of patients with colorectal cancer and increased their 1-year survival rate from 63.4% to 74.3% (Hurwitz *et al*, 2004). However, many patients are still suffering and dying from the disease, and development of additional molecular-targeted anti-cancer drugs is a matter of pressing concern for public health.

These drugs target molecules that are expressed abundantly or exclusively in cancer cells and functioning as an indispensable factor for the growth or survival of cancer cells. For instance, Imatinib (STI571) inhibits several protein kinases such as bcr-abl fusion protein in chronic myelogenous leukaemia, and c-kit in gastrointestinal stromal tumours (O’Dwyer and Druker, 2000). Gefetinib targets the ATP cleft within the EGFR (Wakeling, 2002; Fukuoka *et al*, 2003; Gridelli *et al*, 2003; Kris *et al*, 2003). Trastuzumab is a monoclonal antibody to the HER2/neu receptor, which is overexpressed in ∼30% of breast cancers (Molina *et al*, 2001). These drugs strikingly suppressed the growth of tumour cells and showed minimum cytotoxic effect in normal cells. Therefore, for the development of molecular-targeted anti-cancer drugs pinpointing cancer cells, identification of molecules that are expressed abundantly in cancer cells and clarification of their function are essential.

Molecular studies have clarified that multiple-step process has an important function in colorectal carcinogenesis, which involves activation of oncogenes such as *K-ras*, and inactivation of tumour suppressor genes such as *p53* and *APC*. In addition to these genetic changes, alteration of gene expression is involved in the carcinogenesis. Epigenetic alterations including aberrant DNA methylation and/or histone modification have been recently shown to participate in some of the deregulated gene expression. To unveil the molecular mechanisms of colorectal cancer and discover target molecules for the development of novel anti-cancer drugs, we analysed global gene-expression profiles of colorectal tumours by cDNA microarray analysis representing 23 040 genes (Lin *et al*, 2002). These efforts have identified a number of genes, which are frequently either up-regulated or down-regulated in the tumours compared with the corresponding non-cancerous tissues. Among the list of genes up-regulated in the tumours, we found a gene termed as MRG-binding protein (*MRGBP*), with an approved symbol of chromosome 20 open reading frame 20 (*C20orf20*), which was identified as a component of TRRAP/TIP60 histone acetyltransferase complex and shown to bind directly to MRG15 and MRGX proteins (Cai *et al*, 2003). In this report, we show, for the first time, that MRGBP expression was frequently elevated in colorectal cancer, and that it has an important function in the growth of cancer cells. These findings should contribute to a better understanding of colorectal tumourigenesis, and may serve as a starting point for the development of novel strategies for prevention and treatment of colorectal cancer.

## Materials and methods

### Cell lines and tissue specimens

A human embryonic kidney cell line, HEK293, a monkey kidney cell line, COS7, and human colon cancer cell lines, SW480 and HCT116, were obtained from the American Type Culture Collection (Manassas, VA, USA). All cells were grown in monolayers in appropriate media as follows: Dulbecco's modified Eagle's medium for HEK293 and COS7, McCoy's 5A medium for HCT116, and Leibovitz's L-15 for SW480. All media were supplemented with 10% fetal bovine serum and 1% antibiotic/antimycotic solution (Sigma, St Louis, MO, USA). All colorectal cancer tissues and corresponding non-cancerous tissues were obtained with informed consent from surgical specimens of patients who underwent surgery.

### Isolation of RNA and quantitative PCR

Total RNA was extracted with RNeasy kit (Qiagen, Valencia, CA, USA) according to the manufacturers’ protocols. One microgram of total RNA was reversely transcribed for single-stranded cDNA using oligo(dT)_12−18_ primer (GE Healthcare, Buckinghamshire, UK) with Superscript II reverse transcriptase (Invitrogen, Carlsbad, CA, USA). Quantitative PCR was carried out using the LightCycler 480 System (Roche Diagnostics, Indianapolis, IN, USA). The probes and primers for *MRGBP* and hypoxanthine phosphoribosyltransferase1 (*HPRT1*) are as follows – *MRGBP*: forward, 5′-GGAGGAGACAGTGGTGTGG-3′, reverse, 5′-CATGTGGAAGTGTCGGTTCA-3′, and probe, Universal ProbeLibrary #39 (Roche Diagnostics); *HPRT1*: forward, 5′-TGACCTTGATTTATTTTGCATACC-3′, reverse, 5′-CGAGCAAGACGTTCAGTCCT-3′, and probe, Universal ProbeLibrary #73 (Roche Diagnostics).

### Northern blot analysis

HEK293 cells transfected with pCAGGS-HA-bromodomain containing 8 (BRD8) and/or pcDNA-Myc/His-MRGBP were harvested at the indicated time points after transfection. After purification of RNA, 1 *μ*g of poly(A) RNA was separated on a 1% agarose gel containing formaldehyde and transferred to a nylon membrane. The blot was hybridised with ^32^P-labeled PCR product of *MRGBP* or *β-*actin cDNA. Human multiple-tissue northern blots were obtained from BD Biosciences (Palo Alto, CA, USA), and analysed according to the instructions of the manufacturer. The blots were autoradiographed with intensifying screens at −80°C for 5 days.

### Knockdown of endogenous MRGBP expression by RNA interference

Plasmids expressing short hairpin RNA (shRNA) to *MRGBP* (psiH1BX-MRGBPs), *BRD8* (psiH1BX-BRD8), or *EGFP* (psiH1BX-EGFP) were prepared as described earlier (Shimokawa *et al*, 2003). Briefly, psiH1BX-MRGBPs and psiH1BX-BRD8 were constructed by the cloning of double-stranded oligonucleotides into psiH1BX vector and the target sequences of synthetic oligonucleotides for *MRGBP* shRNAs were as follows: 5′-GAGAAUUUGUAGCGGUUAU-3′ for shMRGBP#1, 5′-GUGACAUGGAUUAGCGCUA-3′ for shMRGBP#2, 5′-ACAAAGUCCUGACCGCAAA-3′ for shMRGBP#3, 5′-GGGAGAAGUGGUGGAAACU-3′ for shBRD8. To evaluate the knockdown effect on MRGBP and BRD8, SW480 and HCT116 cells were transfected with these shRNA constructs using Nucleofector kit (Amaxa, Gaithersburg, MD, USA), and western blotting was performed. For cell proliferation assay, psiH1BX-MRGBPs, psiH1BX-BRD8, or psiH1BX-EGFP were transfected into SW480 and HCT116 cells using FuGENE6 (Roche Diagnostics) according to the manufacturer’s protocol. Transfectants were selected in appropriate concentration of Geneticin (SW480: 1.25 mg ml^–1^, HCT116: 0.7 mg ml^–1^ for 7–9 days), and the viable cells were measured by WST-8 assay (Dojindo, Kumamoto, Japan). Control (ON-TARGET*plus* Non-Targeting pool, Dharmacon, Lafayette, CO, USA) and a mixture of four MRGBP-specific On-Targetplus siRNA oligos (5′-GAGAAUUUGUAGCGGUUAU-3′, 5′-GUGACAUGGAUUAGCGCUA-3′, 5′-ACAAAGUCCUGACCGCAAA-3′, and 5′-CAGGGAAAACCUCGGAUUA-3′) were also used for the functional analysis.

### Flow cytometry

Cultured colorectal cancer cells were transfected with control or *MRBGP* siRNA (Dharmacon) for 48 h. For analysis of cellular DNA content, transfected cells were collected and fixed with 70% ethanol, and then kept at −20°C before use. Cells were incubated with 2 mg ml^–1^ RNase A at 37°C for 30 min and stained with propidium iodide (PI) at room temperature for 30 min. Assessment of apoptosis by annexin V and PI double staining was performed using Annexin V-FITC Apoptosis Detection kit (Medical & Biological Laboratories, Nagoya, Japan). Cellular DNA synthesis was evaluated by incorporation of 5-ethynyl-2′-deoxyuridine (EdU) using Click-iT EdU Flow Cytometry Assay kit (Invitrogen). Briefly, transfected cells were cultured in media containing 10 *μ*M EdU for 30 min. The incorporated EdU and total DNA were stained with Alexa448-conjugated azide and 7-amino-actinomycin D (7-AAD), respectively. Subsequently, the cell suspensions were analysed on an FACSCalibur (Becton Dickinson, Franklin Lakes, NJ, USA) using FlowJo software (Tree Star, Ashland, OR, USA).

### Construction of plasmids expressing MRGBP and BRD8

The entire coding region of *MRGBP* and *BRD8* were amplified by RT–PCR using gene-specific primer sets. The primer sequences used for the amplification were 5′-TGTGAATTCGCCATGGGAGAGGC-3′ (forward) and 5′-TAACTCGAGCGTGCGGCGCCGCTT-3′ (reverse) for *MRGBP*, and 5′-ATAGAATTCTCTTCTGTCATGAGAAGTGG-3′ (forward) and 5′-ATACTCGAGTCACTTTTTCATCTTC-3′ (reverse) for *BRD8*. The cDNA products of *MRGBP* and *BRD8* were cloned into an appropriate cloning site of pcDNA3.1-Myc/His (Invitrogen) or pCAGGS-HA vector, respectively. DNA sequences of all constructs were confirmed by DNA sequencing (ABI3730, Applied Biosystems, Foster City, CA, USA).

### Immunohistochemical staining using polyclonal antibody against MRGBP

We prepared histidine-tagged human MRGBP protein in bacteria, and raised rabbit antibodies specific to MRGBP by immunising rabbits with the MRGBP protein. Purification of antibodies was carried out with standard protocols using affinity columns (Affi-Gel 15, Bio-Rad, Hercules, CA, USA). Specificity of the antibodies was examined by immunoblot analysis using whole extracts from cells expressing Myc-tagged MRGBP (data not shown). Immunohistochemical staining was performed using anti-MRGBP polyclonal antibody. Paraffin-embedded tissue sections were subjected to the SAB-PO peroxidase immunostaining system according to the instructions of the manufacturer (Nichirei, Tokyo, Japan).

### Immunoprecipitation and western blot analysis

COS7 and HEK293 cells were transfected with pcDNA-Myc/His-MRGBP, pCAGGS-HA-BRD8, or the combination using FuGENE6. For immunoprecipitation, the cells were lysed in 0.5% Nonidet P-40 buffer (10 mM Tris–HCl pH 7.5, 150 mM NaCl) supplemented with a Protease Inhibitor Cocktail Set III (Calbiochem, San Diego, CA, USA). The whole-cell extract was incubated with anti-Myc (Santa Cruz Biotechnology, Santa Cruz, CA, USA) or anti-HA antibody (Roche Diagnostics), followed by Protein G-Sepharose beads (Invitrogen) at 4°C. Proteins were separated by SDS–PAGE and immunoblot analysis was performed. Horseradish peroxidase-conjugated goat anti-mouse IgG (GE Healthcare) and goat anti-rat IgG (Santa Cruz Biotechnology) served as the secondary antibody for the ECL Detection System (GE Healthcare). To examine the endogenous interaction of MRGBP and BRD8, nuclear extract from SW480 cells was incubated with anti-MRGBP or anti-p120 (BRD8) antibody (Abcam, Cambridge, UK), followed by Protein G-Sepharose beads overnight at 4°C. After washing, these immunoprecipitants were applied for SDS–PAGE. Normal rabbit IgG (Santa Cruz Biotechnology) was used as negative control.

### Immunocytochemical staining

COS7 cells were transfected with pcDNA-Myc/His-MRGBP, pCAGGS-HA-BRD8, or the combination of the two. Twenty-four hours after transfection, the cells fixed with 4% paraformaldehyde were rendered permeable with PBS containing 0.1% Triton X-100. Subsequently, the cells were covered with 3% BSA in PBS to block non-specific hybridisation, and incubated with anti-Myc or anti-HA antibody. The reaction was visualised after incubation with Alexa Fluor 488 anti-mouse or Alexa Fluor 594 anti-rat secondary antibody (Invitrogen). Nuclei were counterstained with 4′,6′-diamidine-2′-phenylindole dihydrochloride.

## Results

### Expression of MRGBP is frequently elevated in colorectal tumours

**Figure 1:**
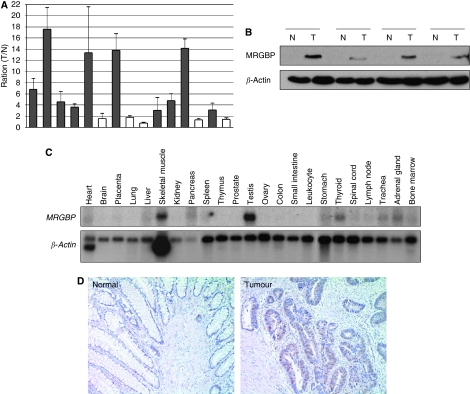
MRGBP is overexpressed in human colorectal tumours. (**A**) Relative expression of *MRGBP* in 15 additional colorectal tumours and the corresponding non-cancerous mucosa was analysed by real-time PCR. Quantity of *MRGBP* was normalised to *HPRT1* expression. The *y* axis indicates the ratio of mean of *MRGBP* expression in tumour to that in normal tissues. The data represents mean±s.d. from three independent experiments. (**B**) Representative western blotting result of MRGBP in normal and tumour tissues from human colon. Expression of *β*-actin served as a control. (**C**) Multiple-tissue northern blot analysis of *MRGBP* in a panel of 23 normal human adult tissues. Expression of *β-actin* served as a control. (**D**) Representative images of immunohistochemical staining of MRGBP in normal and tumour tissues from human colon. Magnification: × 100.

We have earlier compared expression profiles of colorectal cancers with the corresponding non-cancerous colon tissues using cDNA microarray and identified a number of up-regulated genes in the cancer cells (Lin *et al*, 2002). In this study, we investigated a gene termed as *MRGBP* (formally *C20orf20*), because its expression was elevated in 9 out of 11 tumours in our microarray data. Reportedly, MRGBP is a subunit of a transcriptional complex of TRRAP/TIP60. Subsequent quantitative PCR confirmed its elevated expression in 10 out of the additional 15 colorectal tumours compared with their matched non-cancerous mucosa (Figure 1A). Western blot analysis also showed enhanced MRGBP expression in 10 out of the additional 14 tumours examined (Figure 1B). Multiple tissue northern blot analysis using *MRGBP* cDNA as a probe detected a 1.6 kb transcript that was readily detectable in the skeletal muscle, testis, and thyroid, whereas it showed a relatively low level of expression in important normal organs such as heart, brain, lung, liver, and kidney (Figure 1C).

To further evaluate its expression levels, we performed immunohistochemical staining using 27 colorectal cancer tissues. As a result, we observed accumulated MRGBP mainly in the nucleus of cancer cells in 20 out of the 27 tumours. However, non-cancerous epithelial cells in the adjacent mucosa of the tumours did not show accumulation of MRGBP (Figure 1D).

### MRGBP confers growth-promoting effect to cancer cells

**Figure 2:**
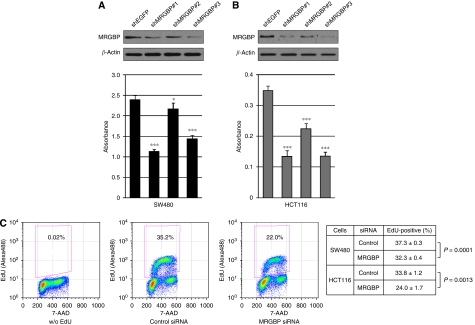
Effect of *MRGBP* shRNA on the proliferation of colorectal cancer cells. (**A**) SW480 and (**B**) HCT116 cells were treated with *MRGBP* shRNAs or *EGFP* shRNA (control) for 48 h, and western blot analysis was performed. Expression of *β*-actin served as a control. Viability of cells transfected with shRNAs was measured by cell proliferation assay kit. The data represents mean±s.d. from five independent transfections. A significant difference was determined by Student's *t-*test; ^*^, *P*<0.05; ^***^, *P*<0.001, *vs EGFP* shRNA-transfected cells. (**C**) SW480 and HCT116 cells were treated with control or *MRGBP* siRNA for 48 h, and then were incubated with 10 *μ*M EdU for 30 min. Representative flow cytometric results of HCT116 cells without EdU incorporation (left) and the cells transfected with control siRNA (middle) or *MRGBP* siRNA (right) were shown. The data represents mean±s.d. from three independent transfections. A significant difference was determined by Student's *t-*test.

To investigate a possible function of elevated *MRGBP* expression in the proliferation of cancer cells, we prepared plasmids that express *MRGBP*- and *EGFP-*specific shRNAs with neomycin resistant gene (psiH1BX-MRGBP1, -MRGBP2, -MRGBP3, and -EGFP). Transfection of SW480 or HCT116 cells with all psiH1BX-MRGBPs significantly reduced the MRGBP expression in the cells, whereas that with control plasmid (psiH1BX-EGFP) did not affect MRGBP expression (data not shown). Cells transfected with psiH1BX-MRGBPs or psiH1BX-EGFP were cultured in media containing appropriate concentration of geneticin, and the number of viable cells was examined at day 7 or 9 after the transfection. As a result, psiH1BX-MRGBPs significantly reduced the number of viable cells compared with control plasmid (Figure 2A and B). To disclose the mechanism(s) underlying the decrease of viable cells by MRGBP knockdown, we investigated induction of apoptosis, cell cycle progression, and DNA synthesis in cancer cells treated with *MRGBP* siRNA. Knockdown of *MRGBP* did not influence significantly on population of apoptotic cells (data not shown). On the other hand, cell cycle analysis showed that treatment of HCT116 and SW480 cells with *MRGBP* siRNA significantly reduced cell population in S-phase compared with control siRNA (35.4±2.0% *vs* 17.5±1.2% in HCT116, *P*=0.0002; 29.4±1.3% *vs* 26.4±0.2% in SW480, *P*=0.016). Consistently, DNA synthesis was suppressed by *MRGBP* siRNA compared with control siRNA (Figure 2C). These results suggested that *MRGBP* might have an essential function in proliferation of colorectal cancer cells through regulation of cell cycle.

### Identification of bromodomain containing 8 as an MRGBP-interacting protein

**Figure 3:**
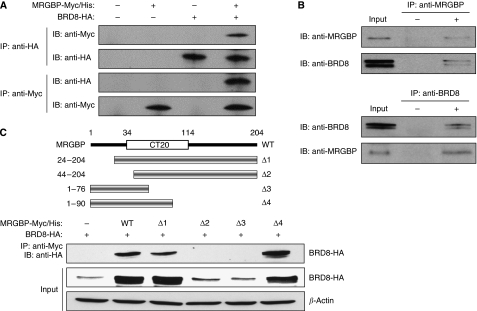
Interaction between MRGBP and BRD8. (**A**) COS7 cells were transfected with pcDNA-Myc/His-MRGBP, pCAGGS-HA-BRD8, or the combination of the two. Extracts from these cells were immunoprecipitated with anti-HA (upper two panels) or anti-Myc antibody (lower two panels). (**B**) Nuclear extract from SW480 cells were immunoprecipitated with anti-MRGBP (upper two panels) or anti-BRD8 antibody (lower two panels). Western blot analysis was performed using the indicated antibodies. (**C**) Interaction of wild type or the deletion mutants of MRGBP with BRD8. HEK293 cells were transfected with HA-tagged BRD8 plasmid and wild type or several deletion mutants of Myc-tagged MRGBP plasmids. Extracts from these cells were immunoprecipitated with anti-Myc antibody and then immunoblotted with anti-HA antibody (upper panel). Expressions of HA-tagged BRD8 and *β*-actin were shown in the middle panel and the lower panel, respectively.

To further investigate the function of MRGBP, we performed yeast two-hybrid screening and identified BRD8 (also known as skeletal muscle abundant protein (SMAP) and p120) as an MRGBP-interacting protein (data not shown). As all 32 positive clones contained the C-terminal region of BRD8, the region was likely to be responsible for the interaction. To confirm the interaction between MRGBP and BRD8, immunoprecipitation assay was performed using plasmids expressing Myc/His-tagged MRGBP (pcDNA-Myc/His-MRGBP) and HA-tagged BRD8 (pCAGGS-HA-BRD8). When COS7 cells were transfected with both pcDNA-Myc/His-MRGBP and pCAGGS-HA-BRD8, immunoprecipitation with anti-HA antibody followed by immunoblotting with anti-Myc antibody showed a single band corresponding to Myc-tagged MRGBP. Consistently, immunoprecipitation with anti-Myc antibody co-precipitated HA-tagged BRD8 (Figure 3A). We further examined endogenous interaction of MRGBP with BRD8 using nuclear extract from SW480 cells. As shown in Figure 3B (upper panels), immunoprecipitation with anti-MRGBP antibody co-precipitated endogenous BRD8. In addition, usage of anti-BRD8 antibody for immunoprecipitation also showed interacting endogenous MRGBP with BRD8 (Figure 3B, lower panels). *BRD8* has been reported to express three transcript variants. Isoform 1 of *BRD8*, which we cloned, represents the predominant transcript. Isoform 2 encodes a protein with a longer and different C-terminal region compared with isoform 1. Although BRD8 isoform 1 contains one bromodomain, isoform 2 contains two. We additionally performed immunoprecipitation experiment using isoform 2 expression plasmid. As a result, isoform 2 also interacted with MRGBP (Supplementary Figure 1A).

### Responsible region of MRGBP for the interaction with BRD8

To address the responsible region of MRGBP for the interaction with BRD8, we prepared various deletion mutants of MRGBP (Figure 3C). Immunoprecipitation and subsequent immunoblot analysis disclosed that wild type and MRGBPΔ1, an N-terminal deletion mutant containing codons 24–204 bound with BRD8. However, MRGBPΔ2 containing codons 44–204 did not associate with BRD8. In addition, MRGBPΔ4, another deletion mutant containing codons 1–90 interacted with BRD8, whereas MRGBPΔ3 containing codons 1–76 did not (Figure 3C). These data indicated that codons 24–90 could be essential for the interaction.

### MRGBP increases BRD8 protein in a post-transcriptional manner

**Figure 4:**
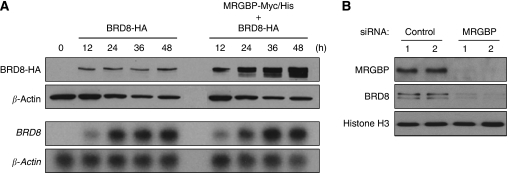
MRGBP increases expression of BRD8 protein in a post-transcriptional manner. (**A**) HEK293 cells were transfected with pcDNA-Myc/His-MRGBP and/or pCAGGS-HA-BRD8. After transfection, the cells were harvested at the indicated time points, and western blot analysis was performed (upper two panels). Expression of *β*-actin served as a control. Simultaneous northern blot analysis using *BRD8* or *β-actin* cDNA as a probe was performed (lower two panels). Expression of *β-actin* served as a control. (**B**) Effect of knockdown of MRGBP on BRD8 expression. HCT116 cells were transfected with *MRGBP*-specific or control siRNA for 72 h. Nuclear extracts were isolated, and western blot analysis was performed using the indicated antibodies. Expression of Histone H3 served as a control.

To examine the levels of BRD8 expression resulting from the interaction with MRGBP, we carried out western blot analysis. Compared with cells expressing exogenous BRD8 alone, the presence of MRGBP markedly enhanced expression of BRD8. The induced BRD8 expression was dependent on the time of transfection, with a continuous increase up to 48 h. In contrast, the levels of BRD8 were unchanged without MRGBP (Figure 4A, upper panels). We also analysed *BRD8* mRNA in the cells by northern blot analysis. As shown in Figure 4A (lower panels), the levels of *BRD8* mRNA were not affected by MRGBP, indicating that the MRGBP-induced BRD8 protein results from post-transcriptional mechanisms. As another experiment showed that MG132, a proteasome inhibitor, greatly enhanced BRD8 protein (Supplementary Figure 1B), the degradation might have an important function in regulating BRD8 expression. Consistent with this view, wild type and mutant MRGBP (Δ1 and Δ4) that associated with BRD8 increased BRD8 expression (Figure 3C). However, mutant MRGBP (MRGBPΔ2 and Δ3) lacking the binding ability with BRD8 did not enhance BRD8 expression (Figure 3C). To confirm the evidence that MRGBP regulates BRD8 expression, we finally knocked down endogenous MRGBP using siRNA. In complete agreement with the result of elevated BRD8 expression by MRGBP, knockdown of MRGBP substantially down-regulated BRD8 expression (Figure 4B). These results identified BRD8 as a novel down-stream target of MRGBP.

## Discussion

We have shown for the first time that MRGBP (C20orf20) is up-regulated in the majority of colorectal cancer, and that its elevated expression is implicated in the proliferation of cancer cells. In addition, we have discovered that MRGBP associates with BRD8. Analysis of TRRAP/TIP60 complex by mass spectrometry identified a number of components including MRGBP (Cai *et al*, 2003). Consistent with our finding, BRD8 was also included in the complex (Cai *et al*, 2003). As all positive yeast clones contained bromodomain in the C-terminal region, bromodomain might be responsible for the binding. Three alternatively spliced forms of BRD8 transcripts have been reported, and all forms include one or two bromodomains at their C-terminal. The predominant variant of transcripts encodes p120 (BRD8 isoform 1), a coactivating factor for thyroid hormone receptor (Monden *et al*, 1997). Interestingly, p120 was also found to interact with PPARγ/RXR heterodimer on PPAR-response elements in the presence of the ligand (Monden *et al*, 1999), suggesting that p120 should be involved in transcriptional regulation. BRD8 isoform 2 contains two bromodomains and has a longer C-terminus compared with p120. Variant 3 encodes SMAP (or BRD8 isoform 3), which was isolated as a highly expressed transcript in skeletal muscle (Nielsen *et al*, 1996). However, the function of SMAP has not been clarified. In addition to isoform 1, we confirmed that MRGBP associates with isoform 2. As isoform 3 shares the same bromodomain with isoform 1, MRGBP should also interact with isoform 3.

In this study, we also examined co-localisation of MRGBP and BRD8 by immunocytochemical staining (Supplementary Figure 2). Consistent with the data of immunohistochemical analysis of MRGBP, exogenous MRGBP protein was accumulated in the nucleus of COS7 cells (Supplementary Figure 2A). On the other hand, BRD8 was mainly localised in the cytoplasm (Supplementary Figure 2B). Interestingly, when COS7 cells were transfected with both plasmids expressing BRD8 and MRGBP, BRD8 accumulated in the nucleus and co-localised with MRGBP (Supplementary Figure 2C). These data implicate that MRGBP alters subcellular localisation of BRD8 and that it increases BRD8 expression in a post-transcriptional manner. As proteasome inhibitor MG132 strikingly augmented BRD8 expression, BRD8 protein is likely to be easily degraded in the proteasome (Supplementary Figure 1B). Taken together, these data suggest that MRGBP may regulate the stability of BRD8. We also found that the interaction of MRGBP with BRD8 is essential for the MRGBP-induced BRD8 accumulation (Figure 3C). Therefore, MRGBP may participate in the shuttling of BRD8 into the nucleus in which proteolysis machinery is inactive. Furthermore, to address the function of BRD8 on cell proliferation, we conducted cell proliferation experiment using *BRD8* shRNA construct. Treatment of HCT116 cells with *BRD8* or control shRNA showed that proliferation of HCT116 cells was significantly reduced by *BRD8* shRNA compared with control shRNA (Supplementary Figure 2D). Therefore, BRD8 may have an important function for cell proliferation as a down-stream target of MRGBP in cancer cells. Although this hypothesis should be investigated in future studies, our findings have uncovered a novel function of MRGBP that is a member of the TRRAP/TIP60 complex.

In summary, the expression of MRGBP is enhanced in the majority of colorectal cancers, and its expression is associated with the growth of cancer cells. Interaction of MRGBP with BRD8 is probably a key for determination of MRGBP function in cancer cells. Our findings will be helpful for the profound understanding of colorectal carcinogenesis and may contribute to the development of novel anti-cancer drugs.
