# Supplementary material for: C20orf20 (MRG-binding protein) as a potential therapeutic target for colorectal cancer
Source: Br J Cancer. 2010 Jan 5;102(2):325–31. doi: 10.1038/sj.bjc.6605500 (PMC2816663; doi:10.1038/sj.bjc.6605500)
Supplement: Supplementary Figures 1 and 2 [file 6605500x1.ppt]

## Slide 1
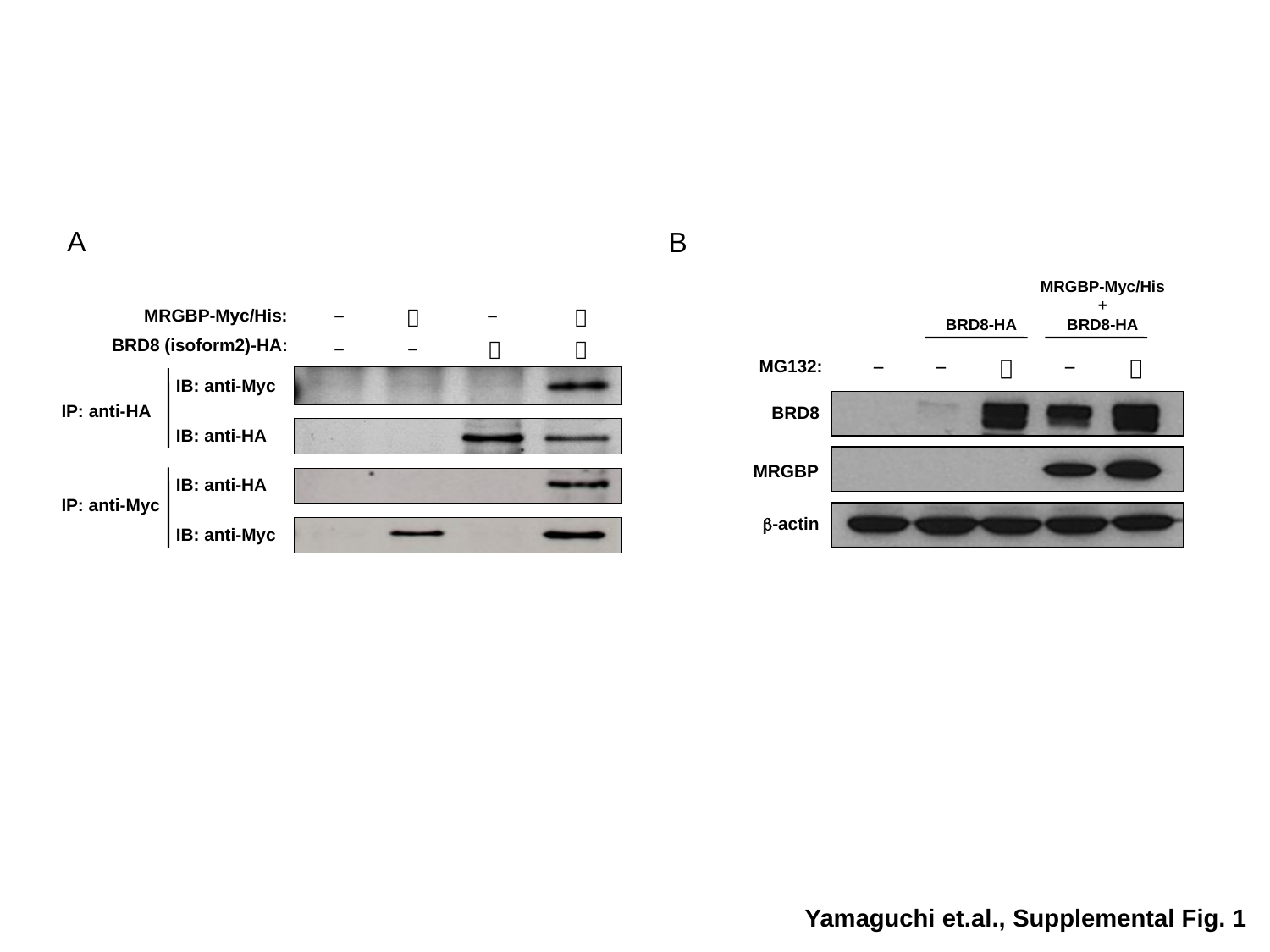

A
B
MRGBP-Myc/His
+
BRD8-HA
MRGBP-Myc/His:
−
＋
−
＋
BRD8-HA
BRD8 (isoform2)-HA:
 ＋
＋
−
−
−
−
＋
−
＋
MG132:
IB: anti-Myc
IP: anti-HA
BRD8
IB: anti-HA
MRGBP
IB: anti-HA
IP: anti-Myc
β-actin
IB: anti-Myc
Yamaguchi et.al., Supplemental Fig. 1

## Slide 2
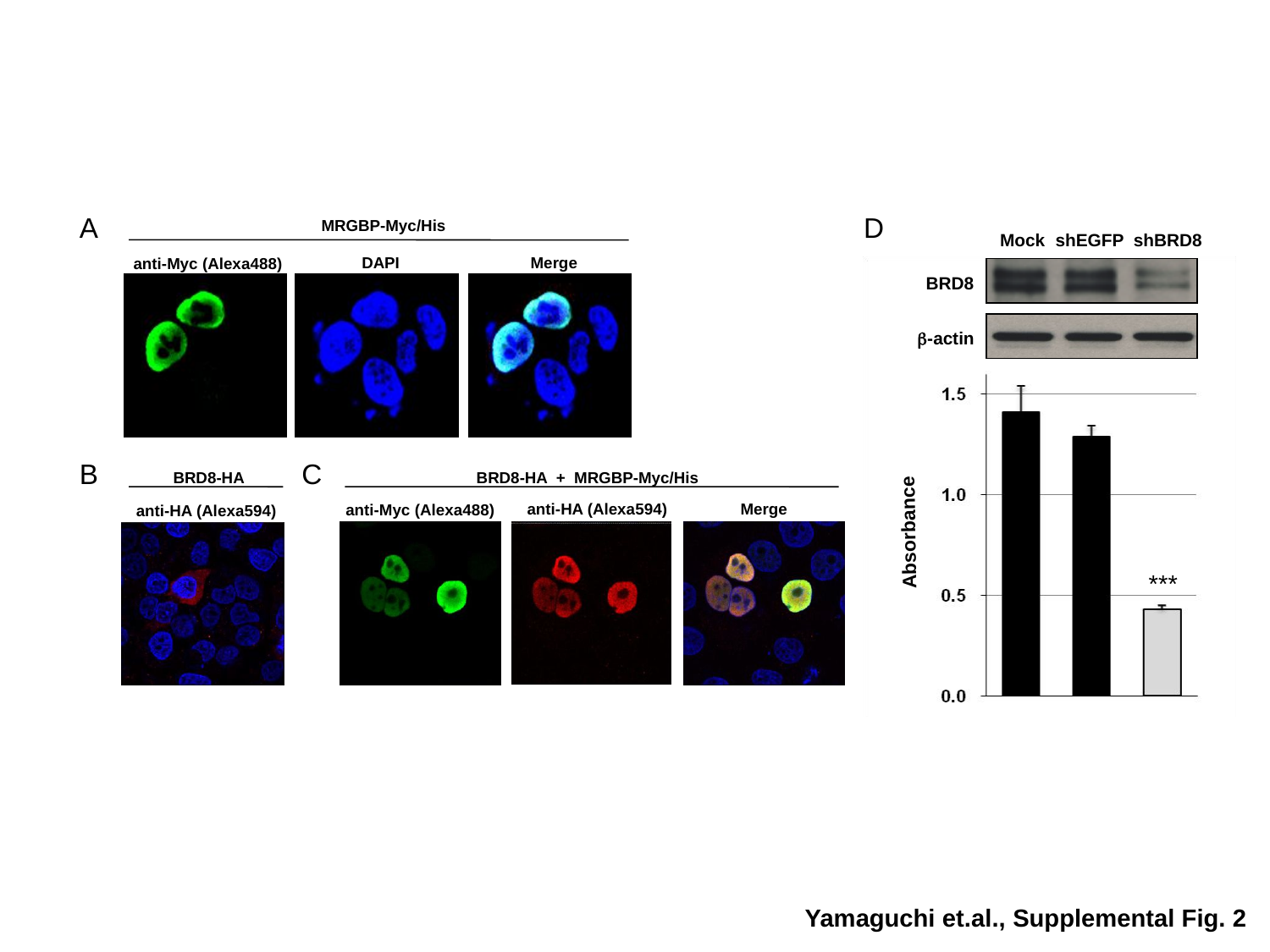

A
D
MRGBP-Myc/His
Mock
shEGFP
shBRD8
DAPI
Merge
anti-Myc (Alexa488)
BRD8
β-actin
B
C
BRD8-HA
BRD8-HA + MRGBP-Myc/His
anti-HA (Alexa594)
Merge
anti-Myc (Alexa488)
anti-HA (Alexa594)
Absorbance
 ***
Yamaguchi et.al., Supplemental Fig. 2
