# Supplementary material for: C20orf20 (MRG-binding protein) as a potential therapeutic target for colorectal cancer
Source: Br J Cancer. 2010 Jan 5;102(2):325–31. doi: 10.1038/sj.bjc.6605500 (PMC2816663; doi:10.1038/sj.bjc.6605500)
Supplement: Supplementary Figures 1 and 2 Legends [file 6605500x2.doc]

**SUPPLEMENTAL FIGURE LEGENDS**

**Supplemental Figure 1** Interaction between MRGBP and BRD8 isoform 2. (**A**) COS7 cells were transfected with pcDNA-Myc/His-MRGBP, pCAGGS-HA-BRD8-2 or the combination. Extracts from these cells were immunoprecipitated with anti-HA (upper two panels) or anti-Myc antibody (lower two panels). (**B**) The cells were transfected with pcDNA-Myc/His-MRGBP and/or pCAGGS-HA-BRD8, followed by treatment with vehicle or MG132, a proteasome inhibitor. Western blot analysis was performed using the indicated antibodies.

**Supplemental Figure 2** (**A**) Subcellular localization of MRGBP. Cells were transfected with pcDNA-Myc/His-MRGBP alone and probed with anti-Myc antibody followed by Alexa488-conjugated anti-mouse IgG secondary antibody (green). (**B**) Cells were transfected with pCAGGS-HA-BRD8 alone and probed with anti-HA antibody followed by Alexa594-conjugated anti-rabbit IgG secondary antibody (red). (**C**) Both pcDNA-Myc/His-MRGBP and pCAGGS-HA-BRD8 were transfected into the cells at the same time. The cells were probed with anti-Myc antibody followed by Alexa488-conjugated anti-mouse IgG secondary antibody (left panel), and with anti-HA antibody followed by Alexa594-conjugated anti-rabbit IgG secondary antibody (middle panel). Nuclei were counter-stained with DAPI. Merged image of Alexa488, Alexa594, and DAPI was shown in the right panel. (**D**) HCT116 cells were treated with psiH1BX-empty vector (Mock), psiH1BX-EGFP, or psiH1BX-BRD8. Viability of cells transfected with shRNAs was measured by cell proliferation assay kit. The data represents mean ± SD from five independent transfections. A significant difference was determined by Student’s *t* test; ***, *P* < 0.001, versus *EGFP* shRNA-transfected cells.
